# Diabetes Mellitus and COVID-19: Associations and Possible Mechanisms

**DOI:** 10.1155/2021/7394378

**Published:** 2021-04-01

**Authors:** Gerui Li, Ze Chen, Zhan Lv, Hang Li, Danqi Chang, Jinping Lu

**Affiliations:** ^1^Department of Geratology, Zhongnan Hospital of Wuhan University, Wuhan 430071, Hubei, China; ^2^Department of Cardiology, Zhongnan Hospital of Wuhan University, Wuhan 430071, Hubei, China; ^3^Department of Geriatrics, Renmin Hospital of Wuhan University, Wuhan 430060, Hubei, China

## Abstract

Coronavirus disease 2019 (COVID-19) is a recently emerged disease with formidable infectivity and high mortality. Emerging data suggest that diabetes is one of the most prevalent comorbidities in patients with COVID-19. Although their causal relationship has not yet been investigated, preexisting diabetes can be considered as a risk factor for the adverse outcomes of COVID-19. Proinflammatory state, attenuation of the innate immune response, possibly increased level of ACE2, along with vascular dysfunction, and prothrombotic state in people with diabetes probably contribute to higher susceptibility for SARS-CoV-2 infection and worsened prognosis. On the other hand, activated inflammation, islet damage induced by virus infection, and treatment with glucocorticoids could, in turn, result in impaired glucose regulation in people with diabetes, thus working as an amplification loop to aggravate the disease. Therefore, glycemic management in people with COVID-19, especially in those with severe illness, is of considerable importance. The insights may help to reduce the fatality in the effort against COVID-19.

## 1. Introduction

The ongoing coronavirus disease 2019 (COVID-19) pandemic caused by severe acute respiratory syndrome coronavirus 2 (SARS-CoV-2) is an enormous global challenge [1]. The clinical spectrum of SARS-CoV-2 infection ranges from mild to critically ill cases, manifesting as asymptomatic infection, mild upper respiratory tract illness, and severe viral pneumonia with respiratory failure and even death [2]. Although most infected people are thought to have a favorable prognosis, chronic diseases commonly seen in elderly people, such as hypertension, diabetes mellitus, cerebral vascular disease, and their susceptibility conditions, may lead to poor clinical outcomes, in time of prepandemic [3]. A meta-analysis showed that the most prevalent comorbidities in people with COVID-19 were hypertension (17 ± 7%, 95% confidence interval (CI) 14–22%) and diabetes (8 ± 6%, 95% CI 6–11%), followed by cardiovascular diseases (5 ± 4%, 95% CI 4–7%) and respiratory system disease (2 ± 0%, 95% CI 1–3%) [4]. Moreover, comorbid diabetes mellitus is thought to increase the risk of COVID-19 infection [5]. Therefore, the high prevalence and incidence of diabetes globally makes this particularly concerning as the COVID-19 pandemic continues to escalate [6].

## 2. Preexisting Diabetes Is Associated with Poor Outcomes of COVID-19

Emerging information suggests that comorbid diabetes in patients with COVID-19 is associated with disease deterioration and even death. Wang et al. [7] reported that, compared to COVID-19 patients who did not receive intensive care, people admitted to the intensive care unit (ICU) were more likely to have preexisting diabetes (8 (22.2%) vs 6 (5.9%), *p*=0.009). In consistent with this, Wu et al. [8] found that comorbid diabetes was more commonly seen in patients who developed acute respiratory distress syndrome (ARDS) than in those who did not develop it (16 (19.0%) vs 6 (5.1%), *p*=0.002). Further bivariate Cox regression analysis found a significant association between preexisting diabetes and a higher risk of developing ARDS (hazards ratio (HR) = 1.13 (1.08, 1.19), *p* < 0.001). By comparing clinical characteristics among COVID-19 patients with and without diabetes, Guo et al. [9] found that people with diabetes were more likely to be severely ill, manifested by a higher incidence of organ damage and hypercoagulability and increased levels of inflammatory factors. Moreover, a retrospective study including 191 COVID-19 patients in Wuhan showed that comorbid diabetes was more frequently seen in nonsurvivors compared to survivors (17 (31%), 19 (14%), *p*=0.0051) [10]. A retrospective case series of 1,591 patients admitted to the ICU with COVID-19 in the Lombardy region found diabetes (17%) is a common underlying medical condition [11]. Moreover, the Italian National Institute of Health reported that 35.5% of the deceased patients with SARS-CoV-2 infection had diabetes [12]. Among 7,162 individuals with laboratory-confirmed COVID-19 reported in the United States from February 12 to March 28, 2020, the prevalence rates for diabetes for patients who were nonhospitalized, hospitalized but not requiring ICU admission, and admitted in the ICU were 6%, 24%, and 32%, respectively [13]. In a case series including 5700 patients hospitalized with COVID-19 in the New York City area, preexisting hypertension and/or diabetes were highly prevalent [14]. Among the dead patients, those with diabetes were more likely to have received treatment in the ICU or invasive mechanical ventilation compared to those without diabetes. Taken together, the above data from different countries suggest that preexisting diabetes is a commonly seen comorbidity and is associated with the severity and mortality of COVID-19. However, it should be noted that none of the abovementioned studies adjusted potential confounders. A meta-analysis demonstrated that the association between diabetes and poor outcomes of COVID-19 was influenced by confounders such as age and coexisting hypertension, and the effect estimate of diabetes was less in older and hypertensive patients [15]. A recent cohort study which retrospectively analyzed 7,337 cases of COVID-19 (among which 952 had preexisting type 2 diabetes) in Hubei Province, China, reported the association between preexisting diabetes and mortality independent of known confounders [16]. In this study, patients with type 2 diabetes required more medical interventions and had significantly higher rates of multiple organ injury and mortality (7.8% vs 2.7%; adjusted HR, 1.49) compared with patients who did not have diabetes. Although this important study addressed the independent association between diabetes and poor outcomes of COVID-19, its retrospective and observational design cannot allow the interpretation of causality. More studies, in particular, large-scale prospective cohort studies and randomized controlled trials (RCTs), are urgently needed to shed light on whether the association between diabetes and COVID-19 outcomes is causative.

## 3. Comorbid Diabetes Is a Predictor for Poor Outcomes of SARS and Middle East Respiratory Syndrome (MERS)

Several studies have investigated the relationship between comorbid diabetes and the two earlier coronavirus infections, the SARS beginning in 2002 and affecting more than 8000 people, mainly in Asia, and the MERS in 2012 affecting more than 2000 people, mainly in Saudi Arabia [17]. It is reported that people with SARS who had comorbid diabetes had higher mortality, and the presence of diabetes was an independent predictor for death from SARS [17, 18]. Moreover, fasting blood glucose (FBG) ≥7.0 mmol/l before steroid treatment was significantly associated with a higher risk of death in SARS patients after adjustment for age and gender (odds ratio (OR) 3.3 (1.4, 7.7), *p*=0.006) [17]. However, a study with over 500 patients hospitalized with SARS in China revealed that, for the majority of patients, hyperglycemia was usually transient and often resolved after discharge [19]. In patients with MERS, diabetes was significantly associated with ventilator support and death and also prolonged MERS-CoV RNA detection in survivors after adjusting for disease severity [20–22]. These results suggest that the presence of diabetes could be considered as a predictor for the progression and outcomes of SARS and MERS.

## 4. Potential Mechanisms for the Association between Diabetes and Poor Outcomes of COVID-19

**Figure 1:**
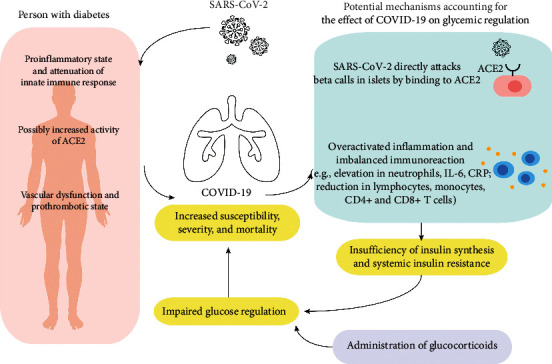
The potential mechanisms for the interaction between COVID-19 and diabetes. Abbreviations: SARS-CoV-2: severe acute respiratory syndrome coronavirus 2; COVID-19: coronavirus disease 2019; ACE2: angiotensin-converting enzyme 2; RAS: renin-angiotensin system; CRP: C-reactive protein.

To date, the underlying mechanisms linking comorbid diabetes and the severity of COVID-19 remain unclear. Aiming to fully characterize this issue and define the best practices for optimum outcomes, it is critical to investigate how diabetes may contribute to disease severity and mortality following SARS-CoV-2 infection. The potential mechanisms for the interactions between diabetes and COVID-19 are illustrated in Figure 1.

### 4.1. Overactivated Inflammation and Imbalanced Immunoresponse

Diabetes mellitus is associated with the proinflammatory state and the attenuation of the innate immune response [4]. Individuals with diabetes have been linked to increased susceptibility to and adverse outcomes associated with infections, especially influenza and pneumonia [23]. Metabolic disorders may impair the functions of macrophages and lymphocytes and thereby lead to low immune function, which predisposes people to disease complications [4]. A rodent study that investigated the effects of diabetes on MERS showed that diabetic mice developed more severe disease and were found to have dysregulated immune responses following MERS-CoV infection, manifested as decreased and delayed recruitment of CD4+ T cells and inflammatory monocytes and macrophages in the lung [24]. In addition to the attenuated overall CD4+ T-cell response, diabetic mice with MERS-CoV infection also showed a more prominent Th17 response with an elevated level of IL-17a, suggesting that an altered cytokine profile could be partly responsible for the increased severity of the disease. In consistent with this, a recent clinical study reported that compared to COVID-19 patients without diabetes, people with diabetes had activated inflammatory response (manifested as elevated levels of neutrophils, serum ferritin, IL-6, C-reactive protein (CRP), and erythrocyte sedimentation rate (ESR)) and suppressed immunity (manifested as markedly decreased levels of lymphocytes) [9]. Notably, a cytokine storm was often observed in fatal cases of COVID-19 and is considered as the main factor that promotes disease progression [9]. These results suggest that COVID-19 patients with diabetes may be more susceptible to overactivated inflammation and imbalanced immunoresponse, which are involved in the pathogenesis of inflammatory storm and rapid deterioration of COVID-19.

### 4.2. Expression and Activity of Angiotensin-Converting Enzyme (ACE) 2

Patients with diabetes usually have concurrent diseases such as hypertension, ischemic heart disease (IHD), and heart failure. Guidelines recommend a renin-angiotensin system (RAS) blocker ACE inhibitor (ACEI) or angiotensin receptor blocker (ARB) for the treatment of hypertension in people with diabetes mellitus, particularly in the presence of microalbuminuria, albuminuria, proteinuria, or left ventricle hypertrophy [25]. However, evidence mainly from animal studies indicates that ACEI/ARB treatment may increase the expression and activity of ACE2 through a feedback mechanism [26, 27]. ACE2 is a homolog of ACE that counterbalances the actions of angiotensin II (Ang II) but is also the known cellular receptor necessary for both SARS-CoV and SARS-CoV-2 infection [28]. As such, concerns have been raised about the possibility that ACEI/ARB could upregulate ACE2 and increase the entry of SARS-CoV-2 into cells, predisposing individuals to infection or increasing the severity of COVID-19 [28]. However, by analyzing the gene expression of ACE2 in 1051 lung tissue samples from the Human Lung Tissue Expression Quantitative Trait Loci Study, Milne et al. found that ACEI use was associated with significantly lower ACE2 expression and ARBs were not associated with altered expression of ACE2 [29]. Nevertheless, a major limitation of this study is that it analyzed gene rather than protein expression. Accumulating evidence also supports the idea that ACE2 might have a dual role in COVID-19, as ACE2 counteracts the deleterious effect of the RAS axis [30, 31]. ACE2 cleaves the last amino acid of Ang II and increases the generation of Ang 1–7, a peptide that acts on the Mas receptor and exerts a vasodilatory effect and antioxidative and anti-inflammatory actions [30, 31]. Reddy et al. reported that, in patients with ARDS, survivors had a higher ratio of Ang 1–7:Ang I compared to nonsurvivors, which supports that the activation of ACE2-Ang 1-7-Mas receptor axis benefits patients with ARDS [32]. Therefore, whether ACEIs/ARBs should be continued in the context of the COVID-19 pandemic has been a recent topic of intense debate [33].

Data from a number of observational studies did not show a significant association between ACEIs/ARBs' use and the risk of SARS-CoV-2 infection or poor COVID-19 outcomes, which supports the statement by several societies that ACEIs and ARBs should be continued in the setting of COVID-19 pandemic [34–36]. Mehta et al. conducted a retrospective cohort study including 18,472 patients tested for COVID-19 in the United States and found no association between ACEI or ARB use and COVID-19 test positivity [37]. Reynolds et al. conducted a study consisting of 12,594 patients who were tested for COVID-19 in a large health care network in New York City. After propensity-score matching, the Bayesian analysis demonstrated no positive association between ACEI and ARB use for either test positivity or severe illness [38]. Furthermore, Abajo et al. conducted a case-population study with 1,139 cases and 11,390 population controls in Spain and found that RAS inhibitors did not increase the risk of COVID-19 requiring hospital admission. Intriguingly, subgroup analysis showed a reduced risk of COVID-19 requiring hospital admission among diabetic patients who were users of RAS inhibitors (adjusted OR 0.53, 95% CI 0.34–0.80) [39]. The activity of ACE is high in the lungs in mouse models of diabetes [40]. If this activity also occurs in humans, it may partly explain the increased severity of COVID-19 in diabetic patients and also the protective effects of RAS inhibitors in COVID-19 patients with diabetes observed in Abajo's study [39].

Several lines of evidence suggest a potential effect of diabetes on the increase of ACE2 expression and activity. A recent phenome-wide Mendelian randomization study that analyzed the association between ACE2 expression and disease states showed that type 2 diabetes is causally correlated with elevated ACE2 expression in the lung [41]. Moreover, significantly increased circulating ACE2 activity was found both in people with type 1 diabetes [42] and in mice with experimental diabetes [43]. In addition, increased levels of urinary ACE2 protein and enzymatic activity were observed in individuals with both type 1 diabetes [44] and type 2 diabetes [45], and values for urinary ACE2/creatinine ratio correlated positively with hemoglobin A1C and FBG [46].

Furthermore, several antidiabetic agents have been reported to affect the expression and activity of ACE2 [47]. Animal studies using the diabetic Akita mouse model showed that insulin treatment was able to inhibit renal ADAM-17 (a disintegrin and metalloproteinase-17) expression, which cleaves ACE2, thereby inactivating the enzyme, whereas sulfonylureas and metformin exhibit no interactions with either ADAM-17 or ACE2 [47]. By contrast, pioglitazone was reported to attenuate ADAM-17 activity in human skeletal muscle and upregulate ACE2 in insulin-sensitive tissues in rats [48]. Similarly, liraglutide, a glucagon-like peptide 1 (GLP1) analog, was shown to increase pulmonary and cardiac ACE2 expression in a rat model of type 1 diabetes [49]. Also, sodium-glucose transporter 2 inhibitors (SGLT2i) were reported to enhance the activity of ACE2, which is a possible mechanism of renoprotection with this class of agents [50]. However, whether such findings readily translate to humans and whether the alterations in ACE2 level would, in turn, affect viral load and the outcomes of COVID-19 in a critical way are not known yet. Further clinical trials and mechanistic studies in both preclinical models and humans are warranted to better define the complex interactions between diabetes, RAS network, and SARS-CoV-2 infection.

Besides ACE2, SARS-CoV-2 cell entry is reported to be depending on transmembrane protease serine 2 (TMPRRSS2), a serine protease highly expressed in the lung and gastrointestinal tissues, including stomach, pancreas, liver, and small and large bowel [51, 52]. In cultured human lung cells, treatment with camostat mesylate, a TMPRSS2 inhibitor, could attenuate SARS-CoV-2 infection [53]. Moreover, Ikeda et al. reported that three patients with diabetic nephropathy presented with nephrotic syndrome who demonstrated a significant reduction in urinary protein excretion after the 7th consecutive day of camostat mesilate treatment [54]. Despite this, there is little data investigating the changes in the expression or activity of TMPRSS2 in the context of clinical or experimental diabetes.

### 4.3. Vascular Dysfunction and Thrombotic Complications

Recent clinical data has highlighted that COVID-19 can predispose patients to thrombotic diseases ranging from microvascular thrombosis to venous or arterial thrombosis [55]. Necropsy and postmortem biopsies of decedents with SARS-CoV-2 infection confirmed the presence of macrovascular and microvascular thrombosis in veins, arteries, arterioles, capillaries, and venules in all major organs [56]. An elegant study by Varga et al. has described endothelial cell infection and endotheliitis across vascular beds in COVID-19 [57]. Emerging evidence suggests that SARS-CoV-2 may invade endothelial cells via ACE2, which is expressed on the endothelial cell surface [58]. In subsequent endothelial inflammation, a variety of pathomechanisms, including leukocyte activation, platelet recruitment, thrombin generation, complement deposition, and the initiation of innate and adaptive immune responses, may culminate in immunothrombosis, leading to significant thrombotic complications [58]. Of note, thrombotic complications usually indicate a poor prognosis of COVID-19 and are associated with multiorgan injury and an increased risk of death [58].

It is known that diabetes is associated with microvascular and macrovascular complications [59]. Hyperglycemia and insulin resistance, together with other metabolic abnormalities in type 2 diabetes, impair the vascular wall through a number of events, including oxidative stress, endothelial dysfunction, platelet hyperactivity, and low-grade inflammation. Activation of these events promotes vasoconstriction and thrombus formation, increasing the vascular risk as well as mortality and morbidity rate in diabetic patients [59, 60]. Thus, vascular dysfunction and prothrombotic state in many diabetic patients may significantly increase the risk of thrombotic complications and death once these patients are infected by SARS-CoV-2. Nevertheless, there remains much interest, and an urgent clinical need, to compare the prevalence and incidence of thrombotic complications between COVID-19 patients with and without diabetes, to delineate the underlying mechanisms, and to investigate whether good glycemic control could reduce the incidence of thrombotic complications in patients with COVID-19.

## 5. Potential Mechanisms Underlying Impaired Glycemic Control Induced by COVID-19

Infections are known to result in less well-controlled diabetes. It is reported that about half of the people infected with SARS-CoV-2 had an elevated level of blood glucose [61]. Indeed, the relationship between diabetes and COVID-19 may be bidirectional. The following part will discuss the potential mechanisms of the promotional effect of SARS-CoV-2 infection on impaired glycemic control.

### 5.1. Beta Cell Damage Induced by Viral Invasion in Islets

Although viruses have long been suspected to influence the risk of type 1 diabetes, the role of respiratory viral infections linked to type 1 diabetes remains obscure. A number of factors, such as microbe species, the host's age, and the intensity of beta-cell stress induced by the infection, may affect this association [62–64]. A prospective study in the United States showed no association between parent-reported early childhood respiratory infections and islet autoimmunity [65]. On the contrary, two small European prospective studies reported that respiratory infections are positively correlated with the initiation of islet autoimmunity [66, 67]. Furthermore, the Environmental Determinants of Diabetes in the Young (TEDDY) study consisting of almost 8000 participants has confirmed that recent respiratory infections are associated with an increased risk of islet autoimmunity in young children [64]. Specifically, the number of respiratory infectious episodes (RIEs) among young children within any nine-month period was associated with the subsequent development of islet autoimmunity within the next three months. Common viruses causing respiratory tract infections include enteroviruses, which have been shown to be implicated in the development of type 1 diabetes [68–70] and are often found in the islets of the pancreas in type 1 diabetic patients [11, 12, 71, 72]. Moreover, a large population-based registry study which included 2.5 million individuals in Norway showed that clinically reported pandemic influenza infection was not significantly associated with an increased incidence of type 1 diabetes (adjusted HR 1.19, 95% CI 0.97–1.46). However, this study showed a twofold higher risk of incident type 1 diabetes in the subgroup with laboratory-confirmed pandemic influenza A (H1N1) (adjusted HR 2.26, 95% CI 1.51–3.38) [73]. Taken together, the abovementioned studies suggest that respiratory viral infections may have a role in the pathogenesis of type 1 diabetes, although evidence from further studies is warranted [64].

Investigations of ACE2 expression in human organs confirmed the expression of ACE2 in the pancreas of normal people, and the expression was slightly higher in the pancreas than in the lungs. Moreover, single-cell RNA sequencing data showed the expression of ACE2 in both islets and exocrine glands of the pancreas [74]. Thus, SARS-CoV-2 may bind to ACE2 in the pancreas and lead to direct injury to islets, impairing glycemic control in patients with COVID-19. Indeed, clinical data showed that pancreatic injury occurred in some patients with COVID-19, mainly in those with severe disease. A recent retrospective cohort study consisting of 121 COVID-19 patients found that about 1% to 2% of nonsevere and 17% of severe COVID-19 patients had a pancreatic injury, as manifested by elevated levels of amylase and/or lipase [74]. Moreover, Yang et al. [17, 19] reported that SARS-CoV may directly damage the pancreatic islets through ACE2, resulting in acute diabetes in SARS patients. Therefore, increased attention should be paid to the pancreas in patients with COVID-19, especially in those with severe illness.

### 5.2. Inflammation-Mediated Insulin Resistance

Diabetes occurs in part because of systemic insulin resistance and beta-cell damage induced by the accumulation of activated innate immune cells in metabolic tissues, which results in the release of inflammatory mediators, in particular, IL-1*β* and TNF*α* [75]. Inflammatory cytokines converge on several critical signaling molecules such as Jun kinase (JNK), inhibitor kappa B kinase *β* (IKK*β*), and nuclear factor-*κ*B (NF-*κ*B) to directly suppress insulin action by serine phosphorylation of insulin receptor substrates 1 and 2 (IRS-1 and IRS-2). Further, cytokines activate multiple suppressors of cytokine signaling molecules, which interfere with activating tyrosine phosphorylation of IRS-1 and IRS-2 and target them for proteasomal degradation [76]. Thus, inflammatory mediators could contribute to the onset and progression of insulin resistance. A number of studies have reported that increased levels of inflammatory mediators such as CRP, IL-6, procalcitonin, and serum ferritin were commonly seen among patients with COVID-19, especially in critical or death cases. Thus, the overactivated inflammatory responses in people infected with SARS-CoV-2 may contribute to insulin resistance and the elevated level of blood glucose [7, 8, 10].

### 5.3. Administration of Glucocorticoids

COVID-19 is a newly emerged respiratory infectious disease, and up to now, there are no effective antiviral agents for treatment. Accumulating evidence suggests that a cytokine storm syndrome may occur in a subgroup of patients with severe COVID-19, and the following virally driven inflammatory organ injury may lead to increased mortality [77]. Thus, it is recommended that all patients with severe COVID-19 should be screened for hyperinflammation based on the levels of inflammatory markers to identify the subgroup of patients who may benefit from immunosuppression therapy [77]. Glucocorticoids are the most commonly prescribed immunomodulatory drugs to module immune activities and reduce inflammation in various viral infections including community-acquired pneumonia, severe influenza, and severe SARS and MERS [78, 79]. However, their use in the setting of COVID-19 has been widely debated [80]. Although glucocorticoids have been recommended for treating severe COVID-19 patients in China [81], many guidelines have stated that glucocorticoids are either contraindicated or not recommended for the treatment of such patients [82]. Recent clinical studies have provided supportive evidence for the use of glucocorticoids in patients with COVID-19. An observational study in China [8] reported that methylprednisolone was given to 62 (30.8%) patients with COVID-19, which was associated with a decreased risk of death in the subgroup with ARDS (HR 0.38; 95% CI 0.20–0.72; *p*=0.003). More importantly, a recent controlled, open-label trial consisting of 6,425 patients hospitalized with COVID-19 found that the use of dexamethasone at a dose of 6 mg once daily for up to ten days led to lower 28-day mortality than usual care among the patients who were receiving either oxygen alone or invasive mechanical ventilation at randomization but not among those receiving no respiratory support [82]. These data indicate that glucocorticoids may be considered as a feasible treatment for critical COVID-19 cases.

It is known that glucocorticoid treatment is associated with a variety of common metabolic side effects including diabetes, hypertension, and osteoporosis [78]. A high dose of glucocorticoids may result in impairment of multiple pathways including beta-cell dysfunction, manifested as impaired sensitivity to glucose and ability to release insulin, and insulin resistance in other tissues. Indeed, steroid-induced hyperglycemia is common in hospitalized patients with and without preexisting diabetes mellitus [83, 84]. The reported prevalence of hyperglycemia after steroid administration in patients without a previous history of diabetes ranges from 20% to 50% [85–87]. Xiao et al. [86] have reported that among 95 patients with SARS who received steroid therapy, steroid-induced diabetes occurred in 33 (34.7%) cases, manifested as elevated FBG levels. After adjusting for age and gender, the daily maximal dose of methylprednisolone was shown to be the only predictor for incident diabetes. Moreover, in hospitalized patients with hematologic malignancies receiving steroids, higher dexamethasone-equivalent steroid dose and longer-acting steroids led to a greater degree of hyperglycemia [84]. Hence, treatment with glucocorticoids is likely to pose great challenges for glycemic control in COVID-19 patients both with and without preexisting diabetes.

## 6. Glycemic Management in Patients with COVID-19

Up to now, there have been limited studies to directly address the role of hyperglycemia in the pathogenesis and prognosis of respiratory viral infections [6]. A recent study [8] pointed out that the increased baseline glycemic level was significantly associated with a higher rate of developing ARDS in patients with COVID-19, although the data were not fully adjusted for confounders. The study by Zhu et al. [16] showed that well-controlled blood glucose (glycemic variability within 3.9 to 10.0 mmol/L) was associated with significantly lower mortality compared to patients with poorly controlled blood glucose (upper limit of glycemic variability exceeding 10.0 mmol/L) during hospitalization (adjusted HR 0.14). These results provide clinical evidence linking improved glycemic control to better outcomes in COVID-19 patients who have preexisting type 2 diabetes, although the causal relationship warrants further validation in well-designed RCTs.

For COVID-19 patients comorbid with diabetes, the optimal goal of glucose control and tailored therapeutic strategy should be formulated based on age, coexisting comorbidities, clinical classification, and other risk factors [88]. Several expert recommendations and reviews have discussed in detail glycemic management in patients with COVID-19 [52, 89, 90]. Here, we briefly listed some special considerations on the use of diabetes drugs that should be taken. Although metformin-induced lactic acidosis and SGLT2i-related diabetic ketoacidosis are rare events, these drugs are not recommended to be continued for patients with severe COVID-19 in order to reduce the risk of acute metabolic decompensation [90]. In addition, sulfonylureas are best avoided in hospitalized patients with severe illness due to the increased risk of hypoglycemia [52]. Importantly, for nonsevere COVID-19 patients with diabetes or diabetic out-patients without any symptoms of infection, prophylactically discontinuing these drugs is not recommended. As for dipeptidyl peptidase-4 (DPP-4) inhibitors and GLP1R agonists, currently, there is no adequate evidence to suggest that these agents should be discontinued. Data from further studies are urgently needed [52, 91]. Of note, if drugs are discontinued, the alternative treatment option should be insulin [90]. Indeed, given the multiple stresses associated with COVID-19, most patients will require insulin treatment. An important observation in many COVID-19 cases across different countries is the very high insulin consumption, especially in patients with severe illness. Thus, it is recommended that insulin needs to be managed by intravenous infusion, bolstered by increasing adoption of continuous glucose monitoring [52, 90]. Considerable care is required in fluid balance and potassium balance following initiation of insulin [92].

Furthermore, recovered COVID-19 patients may have long-term metabolic alterations, as has been reported in SARS survivors [93]. Wu et al. recruited 25 recovered SARS patients 12 years after infection and revealed various serum metabolic disorders in these SARS survivors, including hyperlipidemia (68%), cardiovascular abnormality (44%), and abnormal glucose metabolism (60%). Moreover, a large proportion of the SARS survivors reported glucose metabolic disorders, including hyperglycemia, hyperinsulinemia, insulin resistance, and type 1 or 2 diabetes a few years ahead of the study. Further metabolomics analyses identified systemic metabolic alterations in these patients [93]. Thus, careful cardiometabolic monitoring of recovered COVID-19 patients might be necessary [93]. Further follow-up studies are needed to reveal the long-term metabolic sequelae after COVID-19 infection and investigate the effect of diabetes on any possible future complications.

## 7. Conclusion and Future Perspective

Emerging data suggest that preexisting diabetes is associated with the progression and poor outcomes of COVID-19. The underlying mechanisms of this association remain unclear. Proinflammatory state, attenuation of the innate immune response, possibly increased level of ACE2, along with vascular dysfunction, and prothrombotic state in people with diabetes probably contribute to higher susceptibility for SARS-CoV-2 infection and worsened prognosis. On the other hand, activated inflammation, islet damage induced by virus infection, and treatment with glucocorticoids could, in turn, result in hyperglycemia, leading to difficulty in blood glucose control in COVID-19 patients who have comorbid diabetes. Given the potentially devastating effects of hyperglycemia and ketosis on patients, intensive monitoring and insulin therapy to obtain optimal glycemic control may improve the outcomes of people with COVID-19.

Several critical questions remain unanswered. It has been argued that preexisting diabetes may not lead to an increased risk of COVID-19 infection but a rapid disease progression and worse outcomes. It is important to investigate whether diabetic patients are highly prone to COVID-19 infection or poor outcomes of the disease. It also remains uncertain whether the association between poorly controlled blood glucose is causally related to a poor prognosis [94]. Current recommendations are based on expert opinion, awaiting the outcome of RCTs. Further clinical trials are warranted to investigate the efficacy and safety of different classes of antidiabetic drugs as well as RAS inhibitors to provide evidence-based information for the management of COVID-19 patients comorbid with diabetes. COVID-19 pandemic has brought together the medical community to share information and seek solutions for a large number of patients. At present, we are only at the beginning of this journey together. Much more will be learned about this disease in the coming months and years.
